# Self-assembled amyloid fibrils with controllable conformational heterogeneity

**DOI:** 10.1038/srep16220

**Published:** 2015-11-23

**Authors:** Gyudo Lee, Wonseok Lee, Hyungbeen Lee, Chang Young Lee, Kilho Eom, Taeyun Kwon

**Affiliations:** 1 School of Public Health, Harvard University, Boston, MA 02115, USA; 2 Department of Biomedical Engineering, Yonsei University, Wonju 26493, Republic of Korea; 3 School of Energy and Chemical Engineering, Ulsan National Institute of Science and Technology (UNIST), Ulsan 44919, Republic of Korea; 4 Biomechanics Laboratory, College of Sport Science, Sungkyunkwan University (SKKU), Suwon 16419, Republic of Korea; 5 SKKU Advanced Institute of Nano Technology, Sungkyunkwan University (SKKU), Suwon 16419, Republic of Korea

## Abstract

Amyloid fibrils are a hallmark of neurodegenerative diseases and exhibit a conformational diversity that governs their pathological functions. Despite recent findings concerning the pathological role of their conformational diversity, the way in which the heterogeneous conformations of amyloid fibrils can be formed has remained elusive. Here, we show that microwave-assisted chemistry affects the self-assembly process of amyloid fibril formation, which results in their conformational heterogeneity. In particular, microwave-assisted chemistry allows for delicate control of the thermodynamics of the self-assembly process, which enabled us to tune the molecular structure of β-lactoglobulin amyloid fibrils. The heterogeneous conformations of amyloid fibrils, which can be tuned with microwave-assisted chemistry, are attributed to the microwave-driven thermal energy affecting the electrostatic interaction during the self-assembly process. Our study demonstrates how microwave-assisted chemistry can be used to gain insight into the origin of conformational heterogeneity of amyloid fibrils as well as the design principles showing how the molecular structures of amyloid fibrils can be controlled.

Amyloid fibrils that are formed by protein aggregation have recently been reported to play a vital role in the pathogenesis of various diseases ranging from neurodegenerative diseases^1^,^2^,^3^ (e.g., Alzheimer’s disease and Parkinson’s disease) to type II diabetes^4^,^5^ and cardiovascular diseases^6^. In particular, the aggregation of some intact proteins (e.g., prion protein^7^) or protein fragments (formed due to secretase-driven cleavage or partial unfolding^8^) leads to the formation of highly ordered amyloid structures such as amyloid oligomers, amyloid small aggregates, and amyloid fibrils. These ordered, small aggregates, which include pre-fibrillar forms (e.g., oligomers) and short amyloid fibrils, have recently been highlighted because of their toxicity to functional cells^4^,^5^. In addition, the fibrillar structures (i.e., long amyloid fibrils) have been reported to be able to disrupt the cell membrane^9^. This finding suggests that for the effective treatment of amyloid-driven pathologies, it is of great importance to understand how intact proteins or protein fragments are aggregated to form ordered structures such as amyloid fibrils. Despite the important role of protein aggregation on disease pathogenesis, it has not yet been fully understood how the self-assembly process, i.e., the protein aggregation mechanism, governs the molecular structures of protein aggregates such as amyloid fibrils.

In addition to their pathological role, amyloid fibrils have recently been reported to serve as biocompatible and functional materials. For instance, amyloid fibrils deposited onto an inorganic surface can lead to the formation of a biological thin film that is suitable for bacterial growth^10^,^11^. Amyloid fibrils can act as a catalytic scaffold^12^ that enhances a biochemical reaction, and as a transporting or storing agent that contains or transmits genetic information^13^,^14^ and/or hormones^15^. Moreover, amyloid fibrils have recently been employed for developing biomimetic and functional materials whose material properties can be controlled. For example, researchers in Zurich^16^ developed a biomimetic composite material that was synthesized by coupling amyloid fibrils and graphene sheets and showed that the material properties of such a composite material can be tuned by the chemical environment, such as humidity. Moreover, researchers in Cambridge^17^ demonstrated that a thin film made of amyloid fibrils is not only biologically compatible but also mechanically strong such that the mechanical properties of this film are comparable to those of mechanically strong protein materials such as keratin.

To unveil the mechanisms of amyloid-driven disease expression and to develop biomimetic materials whose material properties can be controllable, it is necessary to understand the self-assembly process that governs the formation of amyloid fibrils, particularly their molecular structures. It has recently been reported that amyloid fibrils can exhibit conformational (structural) diversity, which is a key factor in determining their biological functions^3^ and materials properties^18^. For instance, prion infectivity is determined by the molecular structures of prion amyloid aggregates^7^ such as the size of PrP amyloids^19^ and the helical structures of (HET-s) prion amyloids^20^,^21^. The prion strain phenotypes are determined by the molecular structures of Sup35 prion fibrils, which are highly correlated with their fracture toughness^22^. The toxicity of amyloid protein aggregates has recently been found to be related to their molecular structures, such as their length, such that amyloid particles that are <10 nm long are cytotoxic^23^,^24^. Moreover, recent studies^25^,^26^,^27^,^28^ have reported that the material properties of amyloid fibrils, such as their mechanical properties, are determined by the molecular structures of fibrils (e.g., steric zipper pattern^29^). Specifically, the structural feature of amyloid fibrils, such as steric zipper pattern^27^,^28^, helical pattern^26^, and length^25^, is a key design parameter that determines the material properties of amyloid fibrils. Despite these recent findings^7^,^18^,^19^,^20^,^22^,^23^,^24^,^25^,^26^,^27^,^28^,^29^,^30^ of the important role that the structure (conformation) of amyloid fibrils plays in their biological functions and material properties, it is not well understood how the molecular structures and conformational diversity of amyloid fibrils are determined.

To understand how amyloid fibrils with heterogeneous conformations can be formed, we employed microwave-assisted chemistry^31^, which enables the acceleration of biochemical reactions^32^,^33^ (e.g., protein aggregation). We note that although recent studies^34^,^35^ have reported the microwave-based fast synthesis of amyloid fibrils, these studies^34^,^35^ did not provide any insight into how microwave irradiation affects the protein aggregation mechanism and, consequently, the conformational diversity of amyloid fibrils. Here, we use β-lactoglobulin (βlg) as a model protein because when βlg proteins are aggregated to form βlg amyloid fibrils, they exhibit polymorphic structures^34^,^36^,^37^,^38^. The existence of polymorphic structures for βlg amyloid fibrils led us to study whether microwave-assisted chemistry may be able to control the conformations of these amyloid fibrils. In this work, it is shown that microwave-assisted chemistry enabled us to identify the structurally diverse amyloid fibrils by controlling the thermodynamics of self-assembly and to tune the conformations of βlg amyloid fibrils. Our study provides insight into not only how the amyloid fibrils with conformational heterogeneity can be formed but also how we can control the conformation of amyloid fibrils, which is a key factor in governing their biological functions and material properties.

## Results

**Figure 1:**
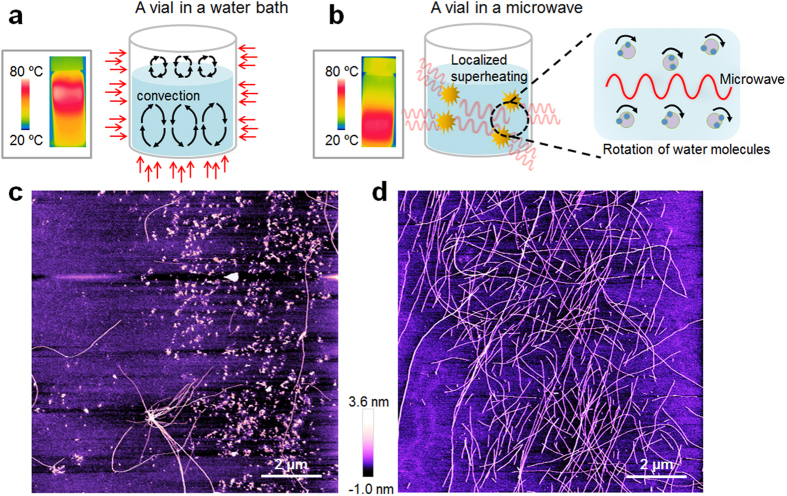
Schematic illustration of (a) classical heating method using a water bath, and (b) microwave-assisted synthesis. Inset figures show the temperature distribution of a protein solution heated for 200 sec by either a classical heating method or microwave-assisted synthesis (**c)** Atomic force microscopy (AFM) image of amyloid proteins synthesized via a classical heating method. **(d)** AFM image of amyloid proteins formed by microwave-assisted chemistry.

Figure 1 illustrates the principle of microwave-assisted chemistry for the synthesis of amyloid fibrils. The microwave-driven acceleration of protein aggregation is attributed to the fact that the temperature distribution of a protein solution heated by microwaves is different from that heated using a classical heating method (Fig. 1a,b). Specifically, microwave irradiation directly increases the temperature of the protein solution because of the microwave-induced rotation of water molecules^39^. By contrast, classical heating methods increase the temperature of air (due to its specific heat) rather than the solution, and heat is then transferred to the solution. In the case of classical heating-based synthesis, both βlg amyloid fibrils and amyloid small aggregates are formed (Fig. 1c), whereas microwave-based chemistry results in the formation of amyloid fibrils with a length of *L* = ~2 μm (Fig. 1d). Notably, microwave-assisted chemistry enabled the rapid synthesis of amyloid fibrils within a few hours (even less than an hour) compared with classical heating methods, which typically require more than several hours.

**Figure 2:**
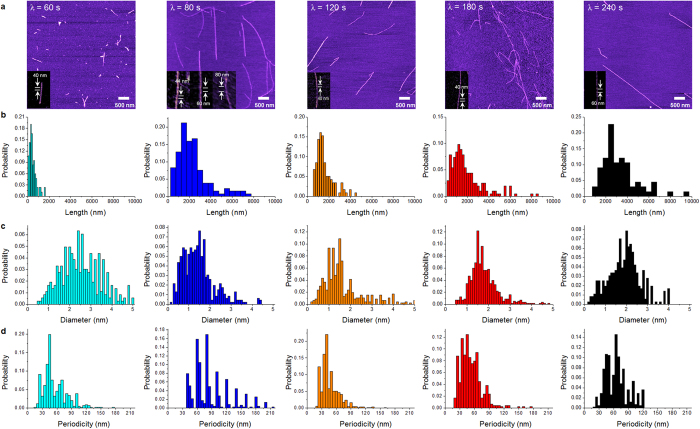
(**a**)AFM images of amyloid fibrils that are synthesized by microwave-assisted chemistry with respect to irradiation time interval, *λ*. **(b**–**d)** Statistical analysis of the length (**b**), diameter (**c**), and helical pitch (**d**) of amyloid fibrils, respectively, that are formed by microwave-based synthesis with a varying time interval, *λ*. It is found that *λ* does not affect the length of amyloid fibrils but critically affects their twisted conformations, particularly their helical pitches.

To manipulate the protein aggregation mechanism, we attempted to transfer microwave to a protein solution based on chronic intermittent irradiation (Fig. S1–S7) by controlling the microwave irradiation time (*τ*), the time interval for irradiation (*λ*), and the number of irradiations (*N*). The optimal condition of microwave irradiation is found to be *τ* = 10 sec such that the optimal energy influx due to wave irradiation is measured as *E* = 0.8 kJ (Supplementary Materials). Based on the optimal energy influx, we found that the structural characteristics of amyloid fibrils depend on the time interval. The irradiation with *λ* = 60 sec results in the formation of short amyloid fibrils with a length of *L* = 0.3 ± 0.2 μm, whereas *λ* ≥ 80 sec leads to the formation of long amyloid fibrils with a length of >1 μm (Fig. 2a).

Now, we characterize the structural features of long amyloid fibrils that are synthesized with *λ* ≥ 80 sec (Fig. 2). It is shown that wave irradiation with *λ* = 80 sec enhances the longitudinal growth of amyloid fibril compared with other *λ* values. In particular, we found that the diameter of the fibrils synthesized with *λ* = 80 sec was *d* = 1.1 ± 0.3 nm, which is smaller than that of other fibrils made with other values of *λ*, and that the aspect ratio of the fibrils made at *λ* = 80 sec is larger than that of other fibrils synthesized with other *λ*’s (data not shown). Here, the aspect ratio of amyloid fibril is defined as the ratio of fibril length with respect to fibril diameter. This result suggests that microwave irradiation with *λ* = 80 sec optimizes the longitudinal growth of amyloid fibrils but does not critically induce their radial growth. In other words, the wave irradiation with *λ* = 80 sec induces a critical temperature (*T*_*C*_ = ~100 °C), at which the longitudinal growth of amyloid fibril is maximized but the radial growth is not optimized. This may occur because at *T*_*C*,_ the unfolding rate of lactoglobulin protein necessary for the longitudinal growth is increased but hydrogen-mediated bonding for the radial growth is hindered^40^. Here, it should be noted that the irradiation with *λ* = 120–240 sec leads to the temperature of a protein solution being estimated as 70–80 °C (<*T*_*C*_). It is shown that as *λ* increases, the diameter of the fibril also increases, which may be ascribed to the reaction time that increases with respect to *λ*. The diameter of the fibril formed based on *λ* ≥ 80 sec is measured in a range of 1.1 to 2.0 nm (Fig. 2c), which corresponds to the diameter of the amyloid protofilament. This suggests that microwave-assisted synthesis allows for the formation of only amyloid protofilaments rather than the multi-stranded fibrils^36^,^41^ that are usually obtained via a classical heating method. More remarkably, the most probable helical pitch of amyloid fibril formed at *λ* = 80 sec is given by *P* = ~80 nm, whereas the other probable helical pitches are estimated to be *P* = 45, 60, and 105 nm, respectively (Fig. 2d). This may be attributed to an unstable stacking mechanism during protein aggregation^40^. However, in the case of *λ* > 80 sec, the distribution of helical pitches for amyloid fibrils is well fitted to a unimodal distribution, which implies a stable protein aggregation resulting in the fibril structure that depends on *λ*.

**Figure 3:**
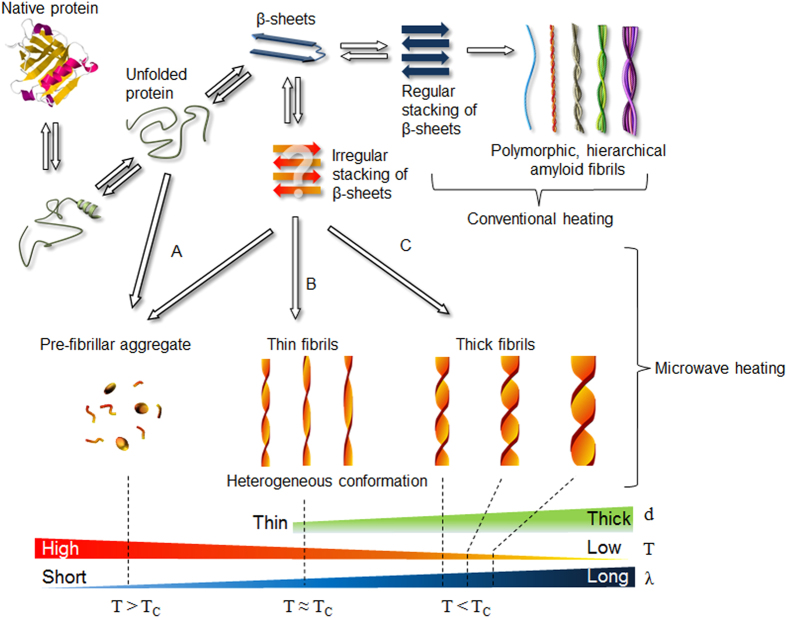
Protein aggregation mechanisms due to microwave irradiation. Pathway (**A**) describes the formation of pre-fibrillar aggregates such as short fibrils and small aggregates that are formed by microwave-assisted chemistry with using *λ* = 60 sec (resulting in *T* > 90 °C). Pathway (**B**) suggests the formation of amyloid fibrils with their diverse twisting conformations due to unstable stacking mechanism when the fibrils were synthesized based on microwave-assisted chemistry with using *λ* = 80 sec. Pathway (**C**) depicts that as *λ* increases (where *λ* > 80 sec), so do the fibril thickness (i.e., diameter) and helical pitch of amyloid fibrils, respectively.

From our observation of the amyloid fibrils formed by microwave-assisted chemistry, we suggest three possible mechanisms of protein aggregations affected by microwaves (Fig. 3). In Pathway A, the wave irradiation with a short time interval (i.e., *λ* < 80 sec) quickly increases the temperature of a protein solution over *T*_*C*_; at this point, the native proteins are rapidly unfolded to be converted to denatured proteins, which results in the formation of small aggregates and short amyloid fibrils. In Pathway B, the wave irradiation with *λ* = 80 sec gradually increases the temperature of a protein solution to *T*_*C*_, when thin amyloid fibrils are formed with conformational heterogeneity. As described above, the wave irradiation with *λ* = 80 sec maximizes the longitudinal growth of amyloid fibrils compared with their radial growth, which results in the high aspect ratio of these fibrils. Pathway C describes wave irradiation with *λ* > 80 sec, which leads to the temperature of a protein solution being ~80 °C, when amyloid fibrils are formed such that their diameter depends on *λ*. We note that unlike a classical heating method^36^,^41^, microwave-based synthesis enables the control of the population of both amyloid fibrils and amyloid small aggregates. Specifically, the microwave-based synthesis with using *λ* = 60 sec (i.e., Pathway A) leads to a predominant population of small aggregates such as pre-fibrillar aggregates, whereas the microwave-assisted chemistry with *λ* ≥ 80 sec (i.e., Pathways B and C) results in the prevalent population of amyloid fibrils with a length of >1 μm (Fig. 2a,b). Our work demonstrates that microwave-assisted chemistry is useful for controlling not only the conformations of amyloid fibrils but also the population of amyloid small aggregates and amyloid fibrils.

**Figure 4:**
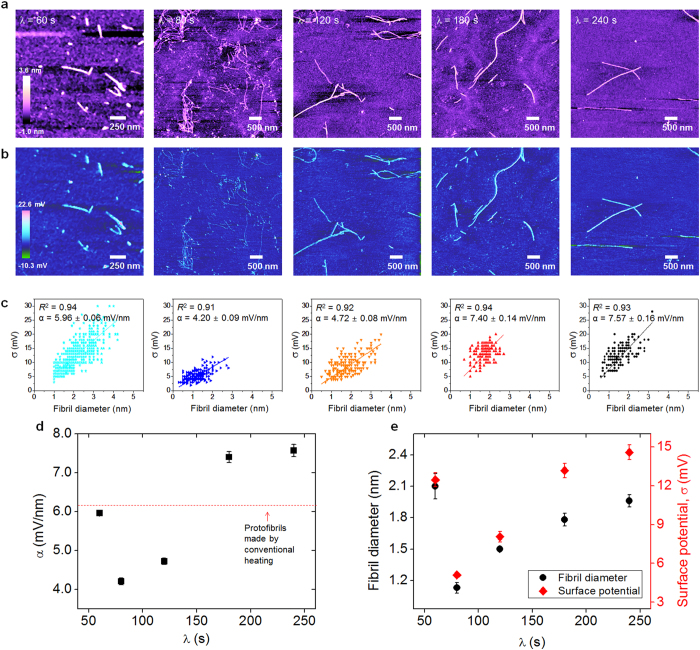
(**a**)AFM images of amyloid fibrils that are synthesized based on microwave-assisted chemistry using different *λ* values. **(b)** KPFM images of the fibrils formed using different *λ* values. **(c)** Distribution of surface charges for amyloid fibril as a function of its diameter. The surface charge potential of an amyloid fibril is almost linearly proportional to its diameter. **(d)** Surface charge density of amyloid fibrils as a function of *λ*. **(e)** The diameter of amyloid fibrils is highly correlated with their surface charge depending on *λ*.

To understand the underlying mechanisms of microwave-induced protein aggregation that results in the formation of amyloid fibrils with their polymorphic structures, we measure the surface charge distribution of amyloid fibrils by using Kelvin probe force microscopy (KPFM) imaging^42^,^43^,^44^, as recent studies^29^,^42^,^45^,^46^ have suggested a critical role for electrostatic interactions in determining the protein aggregation mechanisms. Figure 4 shows the topographic and surface potential images of amyloid fibrils that were synthesized based on microwave-assisted chemistry as well as their conformational heterogeneity. For *λ* = 60 sec, the surface potentials of short amyloid fibril are distributed in a range of 3 to 30 mV with large variation (27 mV) compared with other fibrils synthesized using different *λ* values (Fig. 4c). Here, we note that because the isoelectric point of amyloid fibrils is given by *pI* = ~4.7, the fibril in a solution at pH 2 exhibits a positive surface charge potential^42^,^47^. We measure the surface charge density (*α*), defined as *α*(*x*) = *σ*(*x*)/*d*(*x*), where *σ*(*x*) and *d*(*x*) represent the surface charge potential and diameter of an amyloid fibril, respectively, and a coordinate *x* is defined along the fibril length. We find that except for *λ* = 60 sec, as *λ* increases, the surface charge density also increases (Fig. 4d). Interestingly, the tendency of surface charge density for an amyloid fibril with respect to *λ* is very similar to that of fibril’s diameter depending on *λ*. This finding indicates that microwave irradiation enables the delicate manipulation of protein aggregation mechanisms due to the wave-driven change of electrostatic interaction, which has a critical impact on the radial growth mechanisms of amyloid fibrils (Fig. 4e). Our work shows that microwave-assisted chemistry plays a vital role in determining the twisting conformations of amyloid fibrils and their radial growth (which determines the fibril diameter).

## Discussion

In this work, we first report that microwave-assisted chemistry allows for understanding how heterogeneous conformations of amyloid fibrils can be formed because the microwave affects the thermodynamics of protein aggregation, which is responsible for the formation of amyloid fibrils. We show that the structural characteristics (e.g., helical pitch, diameter, and length) of βlg amyloid fibrils can be controlled by using microwave-assisted chemistry. It should be noted that we study how the structure of βlg amyloid fibrils can be affected by microwave irradiation because recent studies reported the existence of their polymorphic structures^36^,^38^. Our results indicate that microwave-assisted chemistry enables the control of the amyloid fibril structure, which exhibits polymorphic structures. Here, we note that our approach based on microwave-assisted chemistry is only restricted to a specific protein (i.e. β-lactoglobulin) studied in this paper. This study may be extended to other amyloid proteins, which possess the polymorphic structures, for the future work. Our study may provide insight into the conformational heterogeneity of amyloid fibrils for not only further understanding the origin of amyloid-driven pathogenesis (that is dependent on the conformational diversity of amyloid fibrils) but also developing a design principle that is applicable for devising biocompatible and biomimetic materials^11^.

## Methods

### Sample Preparation

The purified β-lactoglobulin (βlg) solution was prepared as previously described^36^,^42^. Briefly, βlg powder (Sigma Aldrich) with 1 wt% is dissolved in pH 2 solution titrated by hydrochloric acid (1 M). More details are provided in the Supplementary Materials. The prepared βlg solution (10 ml) was placed in a microwave (MR-274, LG Electronics). To avoid the evaporation of the solution due to the superheating effect, the vial was hermetically sealed with many layers of paraffin film. To transfer microwave energy to the βlg protein solution, we used chronic intermittent wave irradiation, which is effective in transferring the microwave energy to the solution when the output power of the microwave is high^48^,^49^. Specifically, to delicately control the thermal energy of the protein solution driven by microwave irradiation, we attempted to transfer the wave to thermal energy for a protein solution in a discrete, consecutive manner, which allows for the control of energy influx driven by microwave irradiation. In particular, we controlled the wave irradiation time (*τ*), time interval for irradiation (*λ*), and the number of exposures to wave irradiation (*N*). The details of microwave irradiation conditions that affect the temperature distribution of the protein solution are presented in Fig. S1–S5.

### AFM and KPFM Imaging

A diluted solution containing amyloid fibrils was dropped onto a freshly cleaved mica surface and then rinsed with distilled water or acidic solution three times, followed by nitrogen-based drying. AFM images of amyloid fibrils were obtained in the tapping mode of AFM using a diving board-shaped cantilever tip (TESP, Bruker). For KPFM imaging, the diluted solution was dropped onto a silicon substrate. KPFM imaging was performed using a conductive cantilever tip (SCM-PIT, Bruker). The details of KPFM imaging are reported in our previous works^42^,^43^,^44^. The AFM and KPFM images of amyloid fibrils were analyzed using the Nanoscope Analysis software (Bruker).

## Additional Information

**How to cite this article**: Lee, G. *et al.* Self-assembled amyloid fibrils with controllable conformational heterogeneity. *Sci. Rep.* **5**, 16220; doi: 10.1038/srep16220 (2015).

## Supplementary Material

Supplementary Information

## References

[b1] Dobson C. M. Protein folding and misfolding. Nature 426, 884–890 (2003). 14685248 10.1038/nature02261

[b2] Chiti F. & Dobson C. M. Protein misfolding, functional amyloid, and human disease. Annu. Rev. Biochem. 75, 333–366 (2006). 16756495 10.1146/annurev.biochem.75.101304.123901

[b3] Eisenberg D. & Jucker M. The amyloid state of proteins in human diseases. Cell 148, 1188–1203 (2012). 22424229 10.1016/j.cell.2012.02.022 PMC3353745

[b4] Clark A. & Moffitt J. in Protein Misfolding, Aggregation, and Conformational Diseases (Eds. Uversky V. N. & Fink A. L.) pp. 199–216 (Springer, 2007).

[b5] Höppener J. W. M., Ahrén B. & Lips C. J. M. Islet amyloid and type 2 diabetes mellitus. N. Engl. J. Med. 343, 411–419 (2000). 10933741 10.1056/NEJM200008103430607

[b6] Rapezzi C. *et al.* Transthyretin-related amyloidoses and the heart: a clinical overview. Nat. Rev. Cardiol. 7, 398–408 (2010). 20479782 10.1038/nrcardio.2010.67

[b7] Colby D. W. & Prusiner S. B. De novo generation of prion strains. Nat. Rev. Microbiol. 9, 771–777 (2011). 21947062 10.1038/nrmicro2650 PMC3924856

[b8] Straub J. E. & Thirumalai D. Toward a molecular theory of early and late events in monomer to amyloid fibril formation. Annu. Rev. Phys. Chem. 62, 437–463 (2011). 21219143 10.1146/annurev-physchem-032210-103526 PMC11237996

[b9] Abedini A. & Schmidt A. M. Mechanisms of islet amyloidosis toxicity in type 2 diabetes. FEBS Lett. 587, 1119–1127 (2013). 23337872 10.1016/j.febslet.2013.01.017 PMC4557799

[b10] Barnhart M. M. & Chapman M. R. Curli biogenesis and function. Annu. Rev. Microbiol. 60, 131–147 (2006). 16704339 10.1146/annurev.micro.60.080805.142106 PMC2838481

[b11] Cherny I. & Gazit E. Amyloids: not only pathological agents but also ordered nanomaterials. Angew. Chem. Int. Edit. 47, 4062–4069 (2008). 10.1002/anie.200703133 18412209

[b12] Chapman M. R. *et al.* Role of Escherichia coli curli operons in directing amyloid fiber formation. Science 295, 851–855 (2002). 11823641 10.1126/science.1067484 PMC2838482

[b13] Fowler D. M. *et al.* Functional amyloid formation within mammalian tissue. PLoS Biol. 4, e6 (2005). 16300414 10.1371/journal.pbio.0040006 PMC1288039

[b14] Shorter J. & Lindquist S. Prions as adaptive conduits of memory and inheritance. Nat. Rev. Genet. 6, 435–450 (2005). 15931169 10.1038/nrg1616

[b15] Maji S. K. *et al.* Functional amyloids as natural storage of peptide hormones in pituitary secretory granules. Science 325, 328–332 (2009). 19541956 10.1126/science.1173155 PMC2865899

[b16] Li C., Adamcik J. & Mezzenga R. Biodegradable nanocomposites of amyloid fibrils and graphene with shape-memory and enzyme-sensing properties. Nat. Nanotech. 7, 421–427 (2012). 10.1038/nnano.2012.62 22562038

[b17] Knowles T. P. J. *et al.* Nanostructured films from hierarchical self-assembly of amyloidogenic proteins. Nat. Nanotech. 5, 204–207 (2010). 10.1038/nnano.2010.26 PMC4612398 20190750

[b18] Knowles T. P. J. & Buehler M. J. Nanomechanics of functional and pathological amyloid materials. Nat. Nanotech. 6, 469–479 (2011). 10.1038/nnano.2011.102 21804553

[b19] Silveira J. R. *et al.* The most infectious prion protein particles. Nature 437, 257–261 (2005). 16148934 10.1038/nature03989 PMC1513539

[b20] Govaerts C., Wille H., Prusiner S. B. & Cohen F. E. Evidence for assembly of prions with left-handed β-helices into trimers. Proc. Natl. Acad. Sci. USA 101, 8342–8347 (2004). 15155909 10.1073/pnas.0402254101 PMC420396

[b21] Wasmer C. *et al.* Amyloid fibrils of the HET-s(218–289) prion form a β solenoid with a triangular hydrophobic core. Science 319, 1523–1526 (2008). 18339938 10.1126/science.1151839

[b22] Tanaka M., Collins S. R., Toyama B. H. & Weissman J. S. The physical basis of how prion conformations determine strain phenotypes. Nature 442, 585–589 (2006). 16810177 10.1038/nature04922

[b23] Laganowsky A. *et al.* Atomic view of a toxic amyloid small oligomer. Science 335, 1228–1231 (2012). 22403391 10.1126/science.1213151 PMC3959867

[b24] Liu C. *et al.* Out-of-register β-sheets suggest a pathway to toxic amyloid aggregates. Proc. Natl. Acad. Sci. USA 109, 20913–20918 (2012). 23213214 10.1073/pnas.1218792109 PMC3529048

[b25] Choi B., Yoon G., Lee S. W. & Eom K. Mechanical deformation mechanisms and properties of amyloid fibrils. Phys. Chem. Chem. Phys. 17, 1379–1389 (2015). 25426573 10.1039/c4cp03804e

[b26] Knowles T. P. *et al.* Role of intermolecular forces in defining material properties of protein nanofibrils. Science 318, 1900–1903 (2007). 18096801 10.1126/science.1150057

[b27] Yoon G. *et al.* Mechanical characterization of amyloid fibrils using coarse-grained normal mode analysis. Adv. Funct. Mater. 21, 3454–3463 (2011).

[b28] Yoon G. *et al.* Role of sequence and structural polymorphism on the mechanical properties of amyloid fibrils. PLoS ONE 9, e88502 (2014). 24551113 10.1371/journal.pone.0088502 PMC3925137

[b29] Sawaya M. R. *et al.* Atomic structures of amyloid cross-β spines reveal varied steric zippers. Nature 447, 453–457 (2007). 17468747 10.1038/nature05695

[b30] Yoon G., Kim Y. K., Eom K. & Na S. Relationship between disease-specific structures of amyloid fibrils and their mechanical properties. Appl. Phys. Lett. 102, 011914 (2013).

[b31] de la Hoz A., Diaz-Ortiz A. & Moreno A. Microwaves in organic synthesis: thermal and non-thermal microwave effects. Chem. Soc. Rev. 34, 164–178 (2005). 15672180 10.1039/b411438h

[b32] Kappe C. O. Unraveling the mysteries of microwave chemistry using silicon carbide reactor technology. Acc. Chem. Res. 46, 1579–1587 (2013). 23463987 10.1021/ar300318c

[b33] Kappe C. O., Pieber B. & Dallinger D. Microwave effects in organic synthesis: myth or reality? Angew. Chem. Int. Ed. 52, 1088–1094 (2013). 10.1002/anie.201204103 23225754

[b34] Hettiarachchi C. A., Melton L. D., Gerrard J. A. & Loveday S. M. Formation of β-lactoglobulin nanofibrils by microwave heating gives a peptide composition different from conventional heating. Biomacromolecules 13, 2868–2880 (2012). 22877308 10.1021/bm300896r

[b35] Marek P. *et al.* Efficient microwave-assisted synthesis of human islet amyloid polypeptide designed to facilitate the specific incorporation of labeled amino acids. Org. Lett. 12, 4848–4851 (2010). 20931985 10.1021/ol101981b PMC3052696

[b36] Adamcik J. *et al.* Understanding amyloid aggregation by statistical analysis of atomic force microscopy images. Nat. Nanotech. 5, 423–428 (2010). 10.1038/nnano.2010.59 20383125

[b37] Bolisetty S., Adamcik J. & Mezzenga R. Snapshots of fibrillation and aggregation kinetics in multistranded amyloid β-lactoglobulin fibrils. Soft Matter 7, 493–499 (2011).

[b38] Lara C., Adamcik J., Jordens S. & Mezzenga R. General self-assembly mechanism converting hydrolyzed globular proteins into giant multistranded amyloid ribbons. Biomacromolecules 12, 1868–1875 (2011). 21466236 10.1021/bm200216u

[b39] Bohr H. & Bohr J. Microwave-enhanced folding and denaturation of globular proteins. Phys. Rev. E 61, 4310–4314 (2000). 10.1103/physreve.61.4310 11088227

[b40] Kardos J. *et al.* Reversible heat-induced dissociation of β2-microglobulin amyloid fibrils. Biochemistry 50, 3211–3220 (2011). 21388222 10.1021/bi2000017

[b41] Jung J.-M. *et al.* Structure of heat-induced β-lactoglobulin aggregates and their complexes with sodium-dodecyl sulfate. Biomacromolecules 9, 2477–2486 (2008). 18698816 10.1021/bm800502j

[b42] Lee G. *et al.* Mapping the surface charge distribution of amyloid fibril. Appl. Phys. Lett. 101, 043703 (2012).

[b43] Nam K. *et al.* Aptamer-functionalized nano-pattern based on carbon nanotube for sensitive, selective protein detection. J. Mater. Chem. 22, 23348–23356 (2012).

[b44] Park J. *et al.* Single-molecule recognition of biomolecular interaction via Kelvin probe force microscopy. ACS Nano 5, 6981–6990 (2011). 21806048 10.1021/nn201540c

[b45] Calamai M. *et al.* Nature and significance of the interactions between amyloid fibrils and biological polyelectrolytes. Biochemistry 45, 12806–12815 (2006). 17042499 10.1021/bi0610653

[b46] Moores B. *et al.* Effect of surfaces on amyloid fibril formation. PLoS ONE 6, e25954 (2011). 22016789 10.1371/journal.pone.0025954 PMC3189948

[b47] Konno T. Amyloid-induced aggregation and precipitation of soluble proteins: an electrostatic contribution of the Alzheimer’s β(25−35) amyloid fibril. Biochemistry 40, 2148–2154 (2001). 11329283 10.1021/bi002156h

[b48] Nam K. *et al.* Single-step electropolymerization patterning of a polypyrrole nanowire by ultra-short pulses via an AFM cantilever. Nanotechnology 22, 225303 (2011). 21464524 10.1088/0957-4484/22/22/225303

[b49] Schuster R., Kirchner V., Allongue P. & Ertl G. Electrochemical micromachining. Science 289, 98–101 (2000). 10884233 10.1126/science.289.5476.98

