# Supplementary material for: Self-assembled amyloid fibrils with controllable conformational heterogeneity
Source: Sci Rep. 2015 Nov 23;5:16220. doi: 10.1038/srep16220 (PMC4655422; doi:10.1038/srep16220)
Supplement: Supplementary Information [file srep16220-s1.doc]

***Supplementary Information:***

**Self-assembled amyloid fibrils with controllable conformation heterogeneity**

Gyudo Lee, Wonseok Lee, Hyungbeen Lee, Chang Young Lee, Kilho Eom,* and Taeyun Kwon*

**1. Preparation of protein solution**

βlg protein powder was purchased from Sigma-Aldrich. The powder was dissolved in purified water (100 ml, Gibco), which was titrated by hydrochloric acid (1 M) until the pH of the solution becomes pH 2, in such a way that the concentration of β-lg monomers becomes 1 wt%. The solution was centrifuged at 20,000 rpm for 15 min using a large centrifuge (Hanil Science, Republic of Korea) and then its supernatant (~80 ml) was filtered through a membrane (pore size ~0.22 μm, Millipore, USA). Subsequently, the filtered βlg solution was stored in a few vials such that each vial (with its volume of 25 mL) contains 10 mL of filtered βlg solution. The vial was hermetically sealed, and heated by a microwave under different irradiation conditions as described in Section 2.

**2. Optimal design of microwave irradiation**

We can easily but delicately control the total energy influx into the protein solution by varying wave irradiation conditions such as *τ*, *λ*, and *N.* Microwave irradiation with *τ* = 10 s increases the temperature of the vial up to ~50 °C, while the irradiation with *τ* = 30 or 50 s drastically increases the temperature up to ~100 or ~130 °C (Fig. S1a and S2). The energy influx due to microwave irradiation with *τ* = 10, 30, and 50 s is measured as 0.8, 2.4, and 4 kJ, respectively. As shown in Fig. S4a, with setting *λ* = 1 min, even the few exposure to microwave with *τ* ≥ 30 s critically reduces the volume of a protein solution by ~60%, which may be due to the evaporation of a protein solution in the vial arising from large amount of microwave-driven energy influx (>1 kJ). However, the volume of a protein solution in a vial is almost unchanged even when the vial is exposed several times (*N* = 18) to microwave with *τ* = 10 s and *λ* = 1 min. This suggests that the optimal energy influx due to microwave irradiation, which does not induce a critical change in the volume of a protein solution, is given by *E** = 0.8 kJ. Here, the energy influx due to microwave irradiation with *τ* = 10 s is presented in Fig. S5.

Based on this optimal energy influx, we have delicately controlled the energy influx due to pulse-like microwave irradiation by varying the time interval *λ* in a range of 60 to 240 s, corresponding to the frequency (of energy influx) in a range of 4.0 to 14.3 mHz (Fig. S1b). We measure a change in the mass of a protein solution due to evaporation driven by pulse-like microwave irradiation. It is shown that the change in the mass of a protein solution due to pulse-like microwave irradiation is much less than 0.001 % (Fig. S S4b), which indicates that pulse-like wave irradiation with *τ* = 10 s and frequency of 4.0 to 14.3 mHz is suitable for synthesis of amyloid fibrils.

To gain insight into the thermodynamic behavior affected by pulse-like microwave irradiation, we estimate the temperature of a protein solution (Fig. S3). It is found that the irradiation with *λ* = 60 s abruptly increases the temperature of the solution even up to ~130 °C, while the irradiation with *λ* = 240 s gradually increases the temperature of the solution up to ~74 °C. When *λ* increases, the equilibrium temperature of the solution decreases. It indicates that the heat absorbed into the protein solution due to pulse-like microwave irradiation is critically dependent on the time interval *λ* (Fig. S1c). This clearly elucidates that microwave-assisted chemical synthesis allows us to delicately manipulate the condition of self-assembly process that leads to the formation of amyloid fibrils.

**3. Microwave-based heating vs. classical heating**

In order to understand the difference between microwave-based heating and classical heating, we first take into account the temperature distributions of vial containing the solution, where protein molecules were dissolved, when the vial is exposed to heat via classical heating or microwave-based heating. Fig. S6a-b shows that classical heating increases the air rather than the solution due to the specific heat of air, while the microwave heating directly increases the temperature of solution. This is consistent with Fig. S6c showing that microwave-based heating leads to a fast increase in the temperature of protein solution when compared with classical heating.

Amyloid fibrils that were formed based on either microwave-based heating or classical heating are shown in Fig. S6d and 6e (see an arrow in the figure). As shown in Fig. 6d, the thickness of the fibril synthesized using microwave-based heating is measured between 1 nm and 2 nm, which is comparable to that of amyloid protofilament. On the other hand, the fibril formed by classical heating exhibits the thickness of ~3 to ~4 nm, which indicates that the fibril may be made of two filaments. The AFM image of fibril (that was synthesized from microwave-based heating) deposited onto the mica surface is also shown in Fig. S7.

**4. AFM and KPFM Imaging of Amyloid Fibrils**

Fig. S8 provides the AFM and KPFM images of amyloid fibrils that were synthesized based on microwave irradiation with using different irradiation time intervals. In addition, the AFM height profile and KPFM surface charge profile of the fibrils were also provided in Fig. S8.

**5. Effect of synthesis time on the structure of amyloid fibrils**

In order to understand the effect of synthesis time on the structure of amyloid fibrils, we have analyzed the fibrils that were formed based on microwave-based heating with identical exposure time *τ* and time interval *λ* (i.e. fixing *τ* = 10 sec and *λ* = 180 sec) but varying number of exposure (*N*). The statistical analysis, shown in Fig. S9, suggests that the number of exposure does not critically affect the structural feature of amyloid fibrils, indicating that the key parameter, which determines the structural feature of the fibrils, is the time interval.

**Figure S**
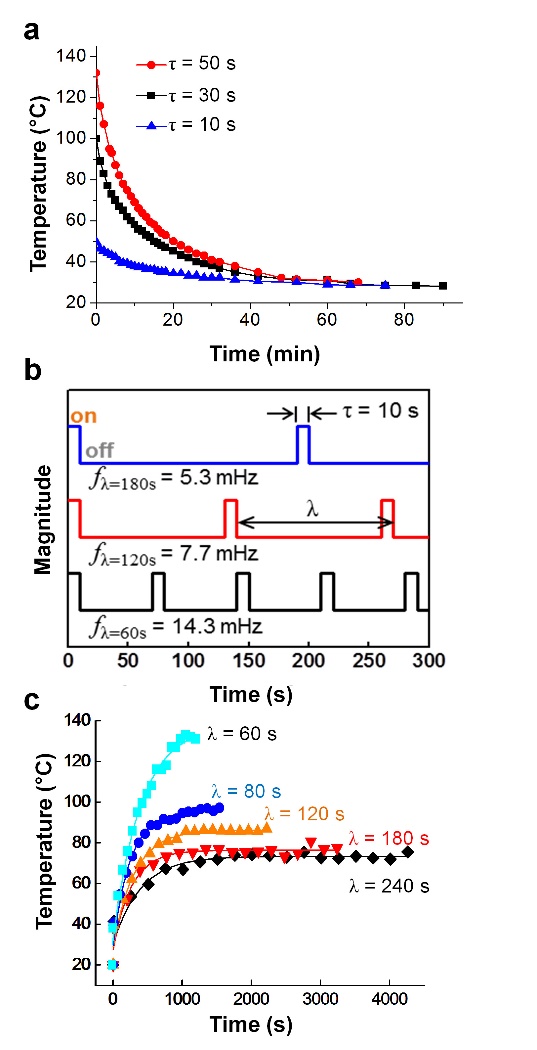
**1.** A strategy of microwave energy transfers using discrete microwave irradiation for thermal energy-dependent formation of amyloid-like fibrils. **(a)** Experimental time-temperature curves for a vial subjected to natural cooling after microwave irradiation with different exposure time *τ* of 10, 30, and 50 s, respectively. **(b)** Condition of microwave irradiations with different time interval *λ* under identical exposure energy based on *τ* = 10 s and *N* = 18. **(c)** Temperature variation curves of protein solution under microwave irradiation with different time interval *λ*.


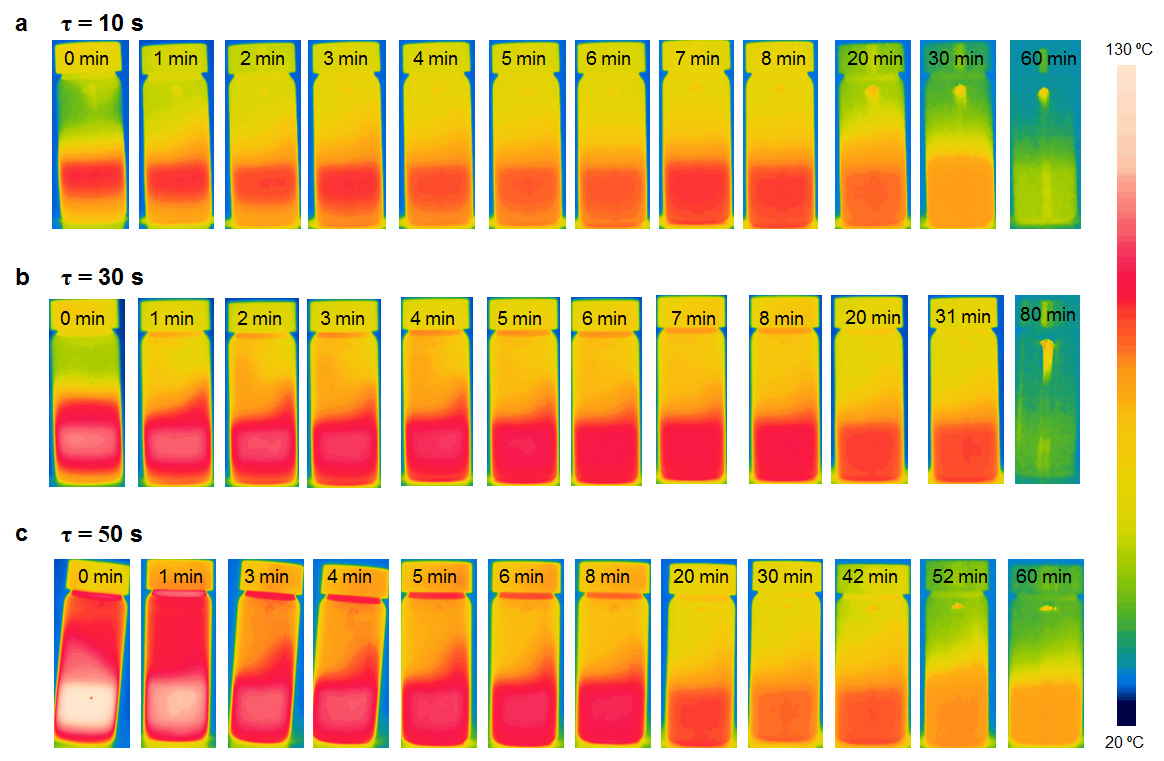


**Figure S2.** Time-lapse thermography of a vial subjected to natural cooling after microwave irradiation with different exposure time *τ* of 10 s (**a**), 30 s (**b**), and 50 s (**c**).


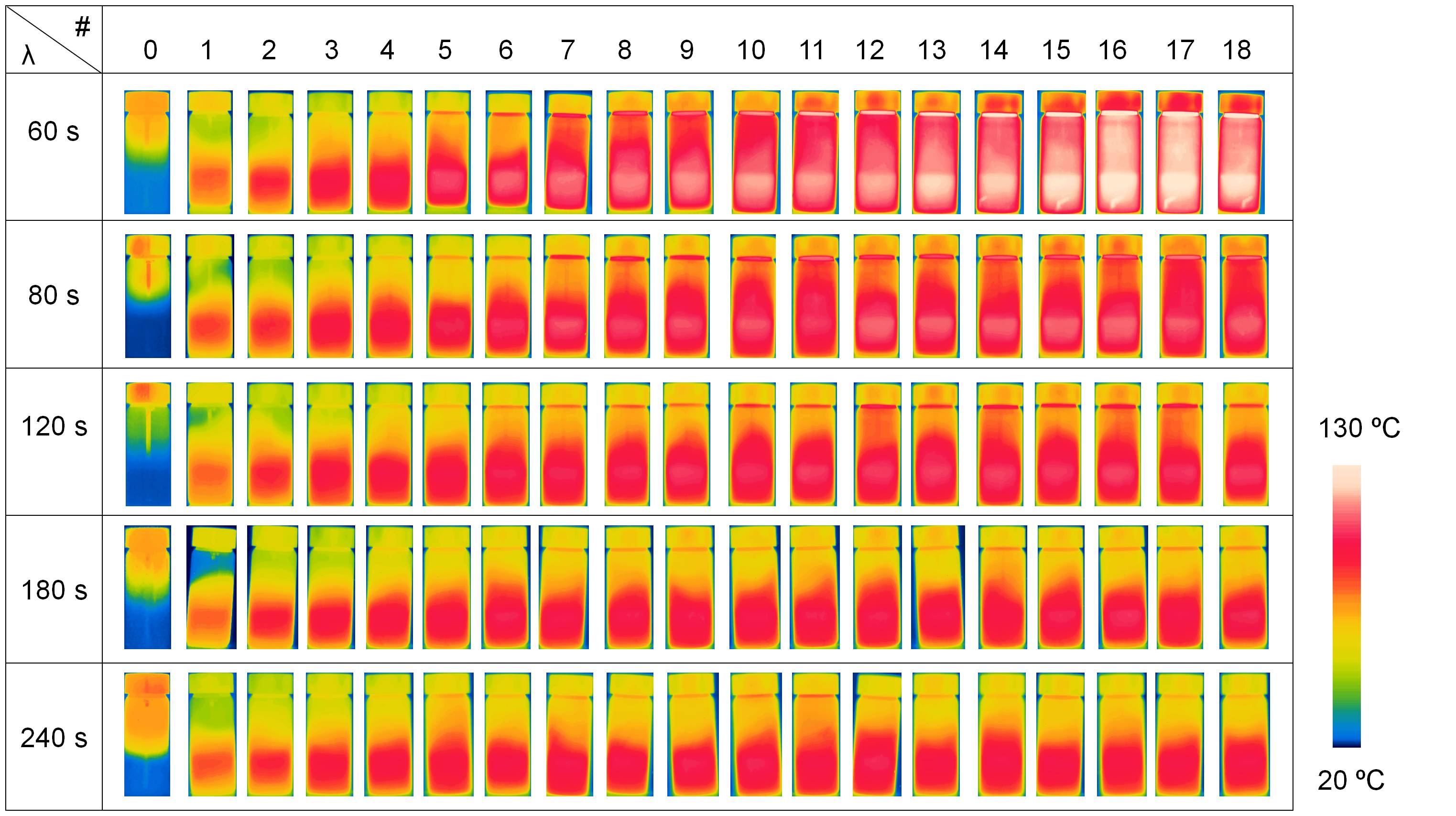


**Figure S3.** Time-lapse thermography of a vial subjected to microwave irradiation with different time interval *λ* and the number of exposure *N* = 1 to 18 with an identical exposure energy of *τ* = 10 s.


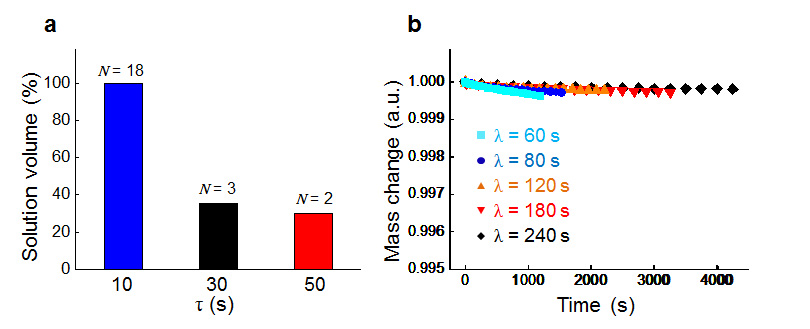


**Figure S4.** **(a)** The change of a volume for a protein solution due to microwave irradiation with different exposure time *τ* and number of exposure *N*. **(b)** Time-dependent mass change of protein solution due to wave irradiations with different time interval *λ*.


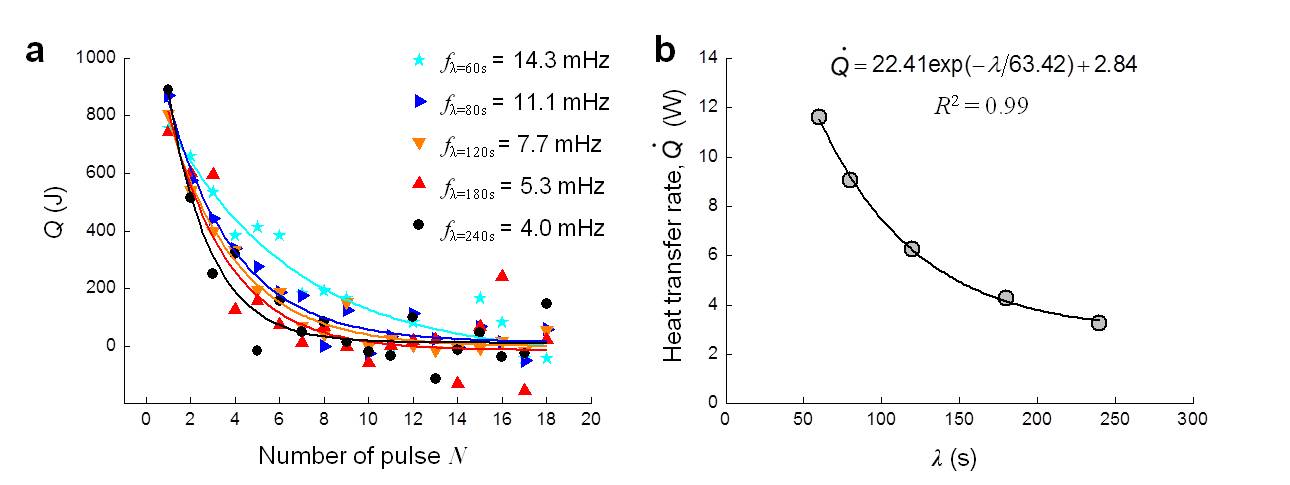


**Figure S5.** Energy influx into protein solution of 10 ml due to wave irradiation. **(a)** Heat energy *Q* per pulse with different frequency *f* is computed from a relation of *Q_N_* = *Cm*Δ*T_N_*, where *N* is the number of wave irradiations, *C* is the specific heat capacity of water, i.e. *C* = 4.1813 Jg^-1^K^-1^, *m* is the mass of water (here, *m* = 10 g), and Δ*T* is the change of temperature of water. The plot indicates that heat energy absorbed into the protein solution due to wave irradiation is exponentially decreasing with the number of pulses because of energy equilibration. **(b)** Calculated average amount of the rate of heat transferred to the solution. $\mathop{Q}\limits^{\bullet} =Qf$, where *f* is applied microwave frequency *f* = 4.0 to 14.3 mHz.

***
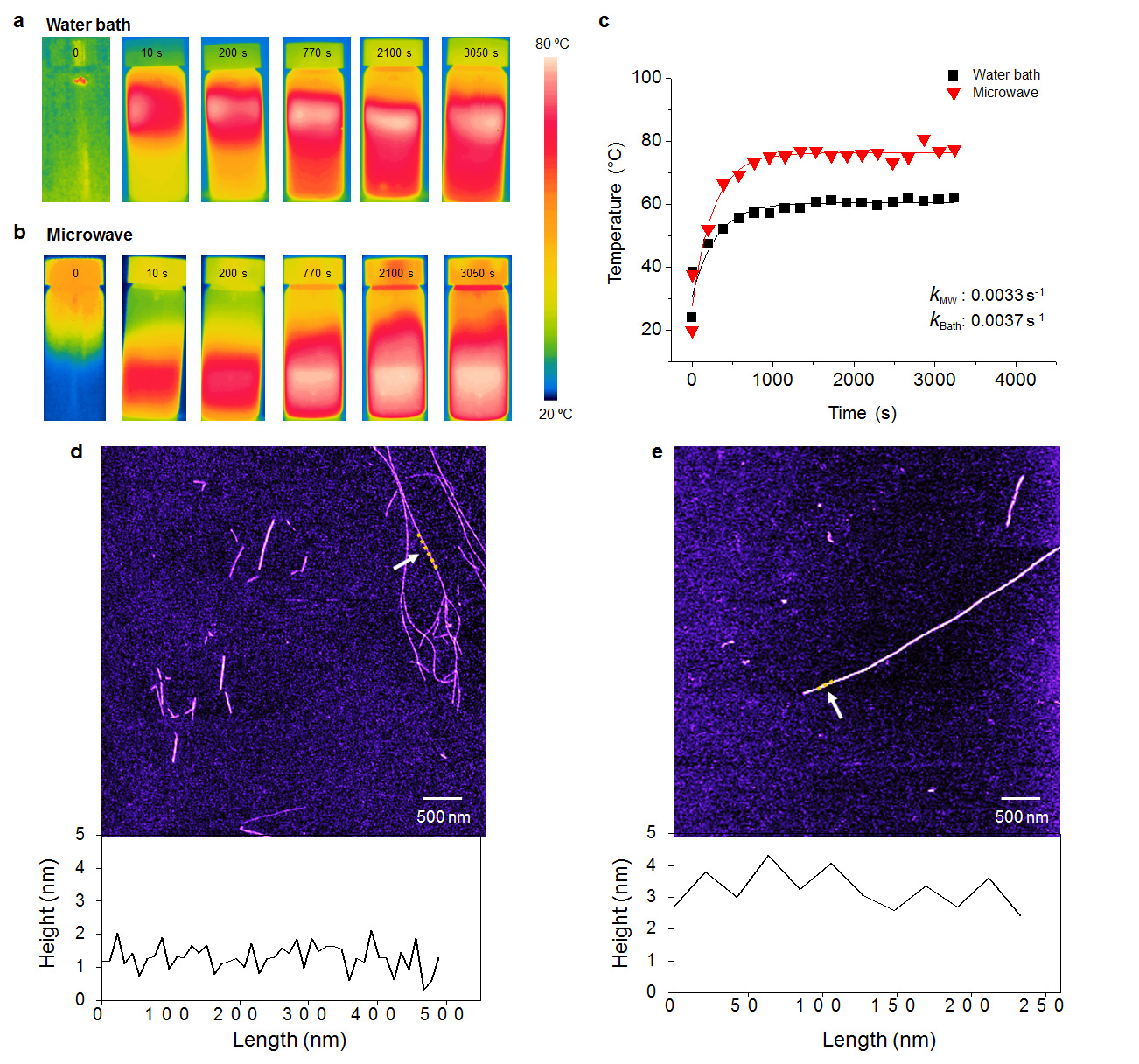
***

**Figure S6.** Temperature distribution of a βlg protein solution based on two heating methods using water bath and microwave irradiation, respectively. All thermal images were recorded in home-built darkroom with using an infrared camera (T620, FLIR, USA). (**a) – (b)** In a classical heating method based on water bath (a), air in the vessel was heated earlier than the solution because of difference between specific heat capacities of air and solution. However, for microwave irradiation-based heating (b), the solution was directly heated by wave irradiation. **(c)** Plot of the average temperature of the vessel as a function of heating time. The curve of time-dependent average temperature for the vessel was fitted to exponential curve. **(d) – (e)** AFM images of amyloid fibrils synthesized (with synthesis time of 1 hr) using microwave irradiation-based heating (d) and water bath-based heating (e). The thickness of the fibril made by wave irradiation-based heating is two times less than that made by classical heating method, which implies that the wave irradiation-based heating leads to the formation of amyloid protofilament (i.e. single filament) while the classical heating method results in mature fibrils that are made of two filaments.


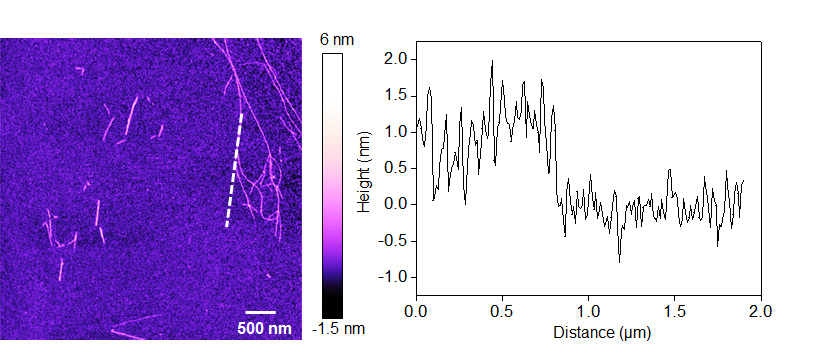


**Figure S7.** AFM height profile of amyloid fibrils on a mica substrate.


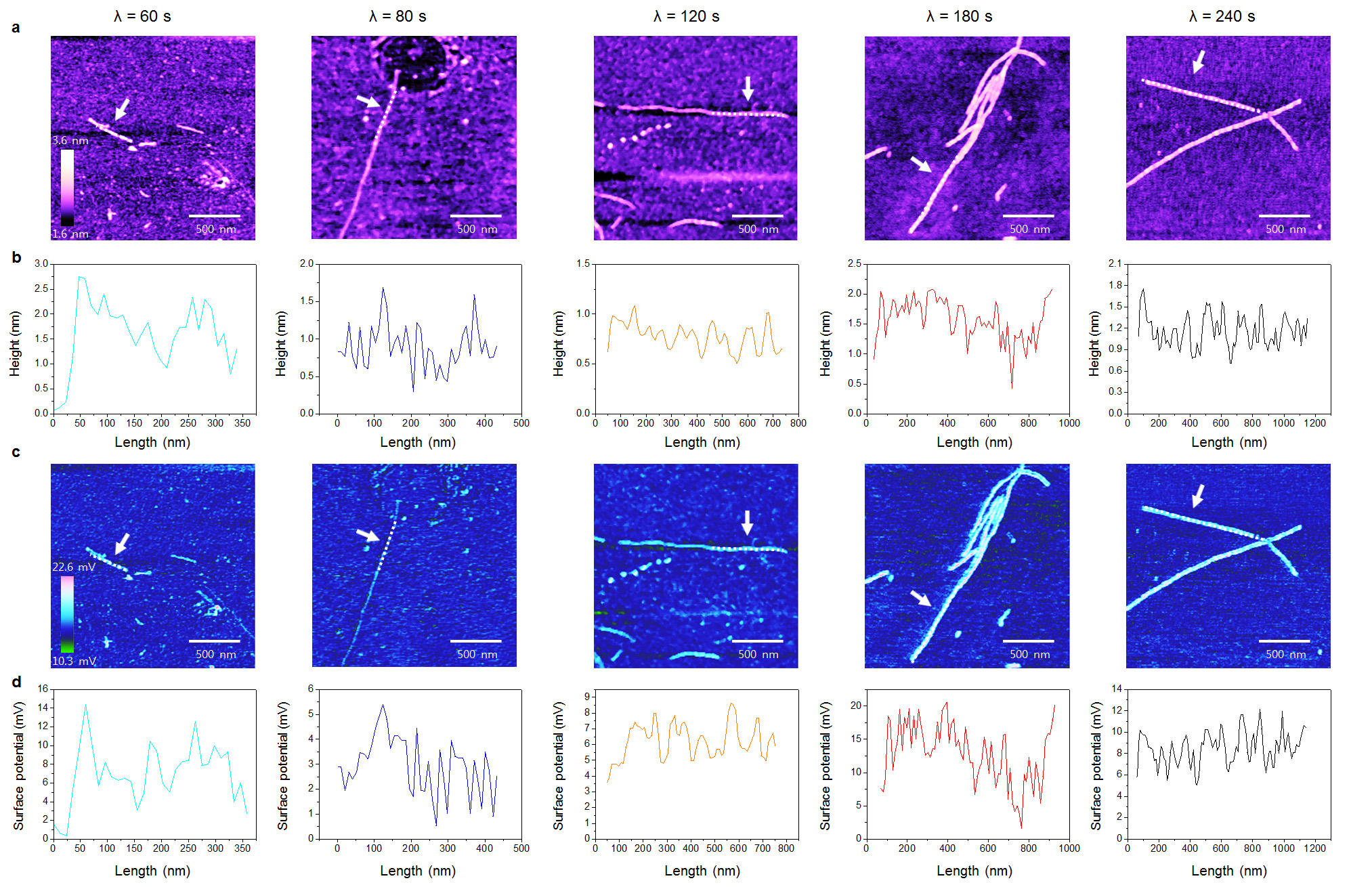


**Figure S8.** **(a) – (b)** Topologic images (a) and the height profiles (b) of βlg amyloid fibrils synthesized using microwave with different time interval *λ* = 60 to 240 s, under an identical exposure time *τ* = 10 s and the number of exposure *N* *=* 18. **(c) – (d)** KPFM images (c) and the surface potential profiles (d) of βlg amyloid fibrils. The section profiles (b, d) were extracted from white dotted line in AFM and KPFM images.


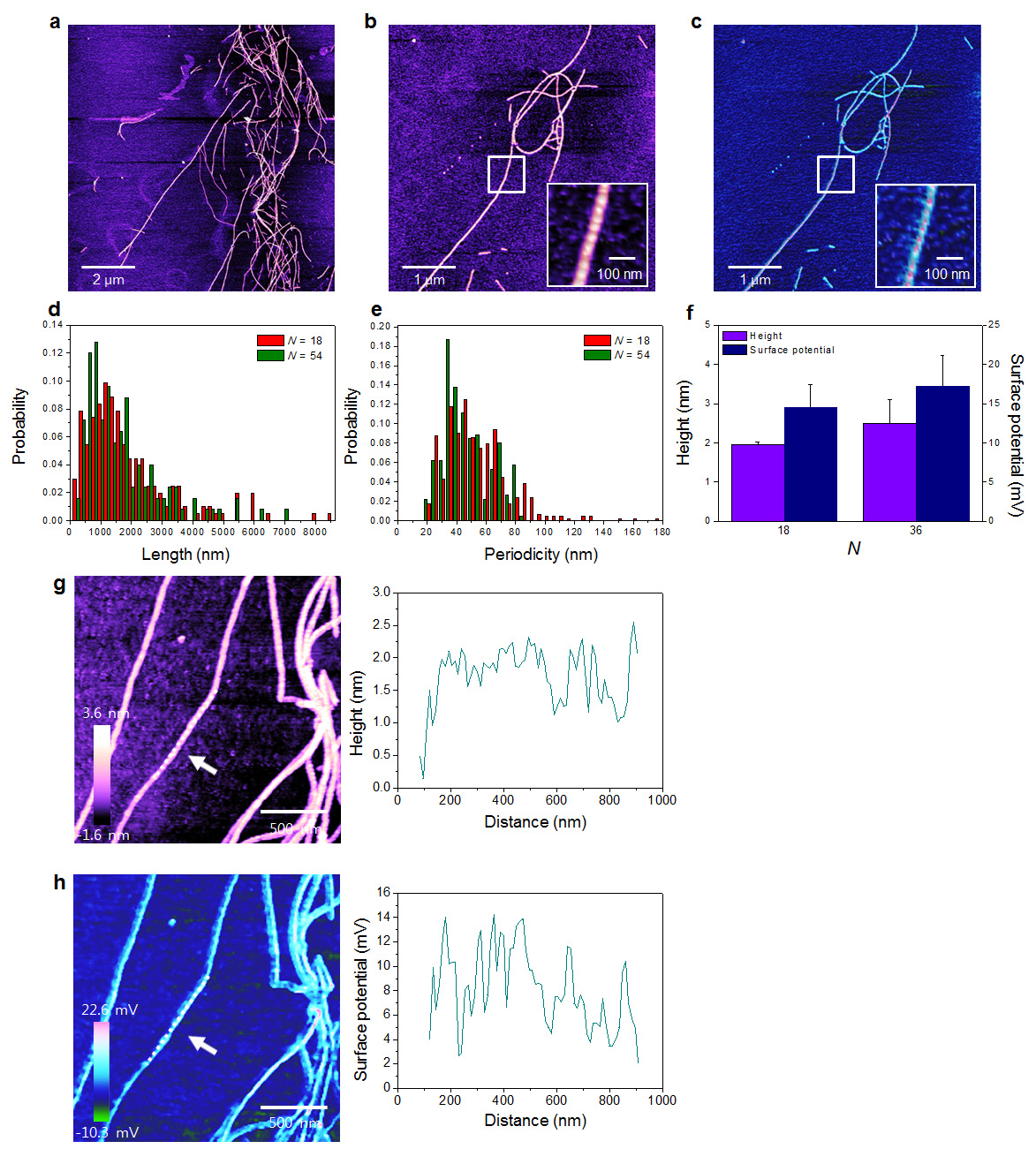


**Figure S9.** Characterization of the structural conformations of amyloid fibrils synthesized using microwave irradiation with the number of exposure *N* = 54 (9 min) for 3 hr under an identical exposure time *τ* = 10 s and the time interval *λ* *=* 180 s. **(a)** AFM topographic image of amyloid fibrils. **(b) – (c)** High-resolution images, i.e. AFM and KPFM images, of amyloid fibrils synthesized using microwave irradiation for 9 min. **(d) – (e)** Probability distributions for the length (d) and helical pitches (e) of amyloid fibrils formed based on wave irradiation for 9 min (i.e. *N* = 54) and 3 min (i.e. *N* = 18), respectively. **(f)** The average AFM heights and surface charge densities of the fibrils synthesized using wave irradiation for 9 min and 3 min, respectively. The results show that the conformations of amyloid fibrils made by wave irradiation for 9 min are similar to those synthesized using wave irradiation for 3 min. **(g)** AFM topographic image with height profile and **(h)** KPFM image with surface potential profile for amyloid fibrils synthesized by microwave irradiation for 9 min (with a condition of *τ* = 10 s; *λ* = 180 s; *N* = 54).
